# Supplementary figures and images for: A monocarboxylate transporter rescues frontotemporal dementia and Alzheimer’s disease models
Source: PLoS Genet. 2023 Sep 21;19(9):e1010893. doi: 10.1371/journal.pgen.1010893 (PMC10513295; doi:10.1371/journal.pgen.1010893)

**A**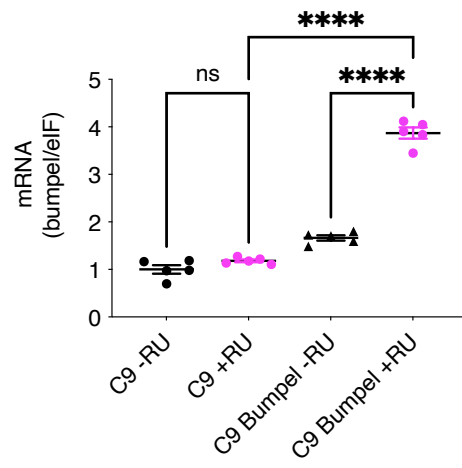**B**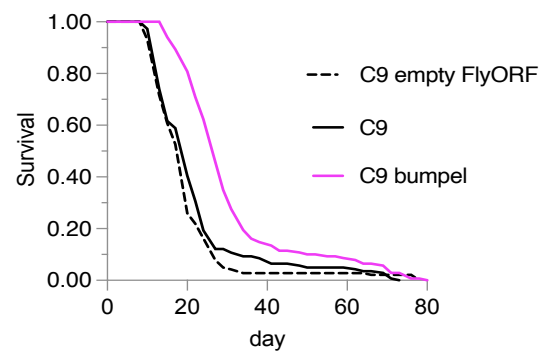

Supplement: S1 Fig — A. Bumpel mRNA levels measured by qPCR, relative to eIF in C9 and C9 bumpel expressing flies (+RU) and controls (-RU). Genotype: UAS-36 (G4C2), elavGS, UAS-36 (G4C2), elavGS/UAS-bumpel. ****p<0.0001 by Šídák’s multiple comparisons test following one way ANOVA B. Lifespan of 36R expressing flies with and without bumpel co-expression. Genotype: UAS-36 (G4C2), elavGS; UAS-36 (G4C2), elavGS/UAS-empty FlyORF; UAS-36 (G4C2), elavGS/UAS-bumpel p = 3.4E-17 for comparison of C9 bumpel to C9 empty flyORF and p = 1.3E-10 for C9 bumpel to C9. (PDF) [file pgen.1010893.s001.pdf]

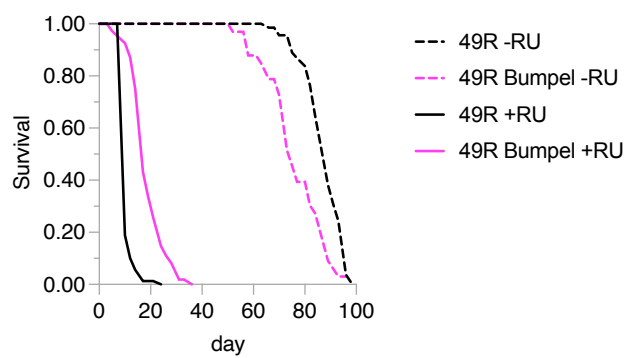

Supplement: S2 Fig — Genotype: UAS-49 (G4C2), elavGS, UAS-49 (G4C2), elavGS/UAS-bumpel p = 6.9E-36 for +RU comparisons. (PDF) [file pgen.1010893.s002.pdf]

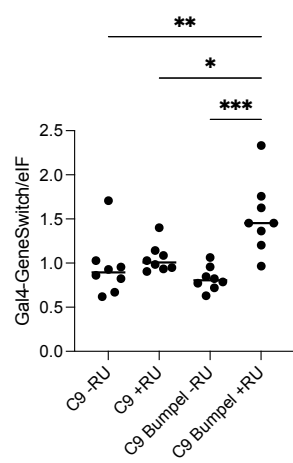

Supplement: S3 Fig — ***p = 0.0002, **p = 0.0022, *p = 0.0142 by Šídák’s multiple comparisons test following one way ANOVA. Genotypes; UAS-36 (G4C2), elavGS, UAS-36 (G4C2), elavGS/UAS-bumpel. (PDF) [file pgen.1010893.s003.pdf]

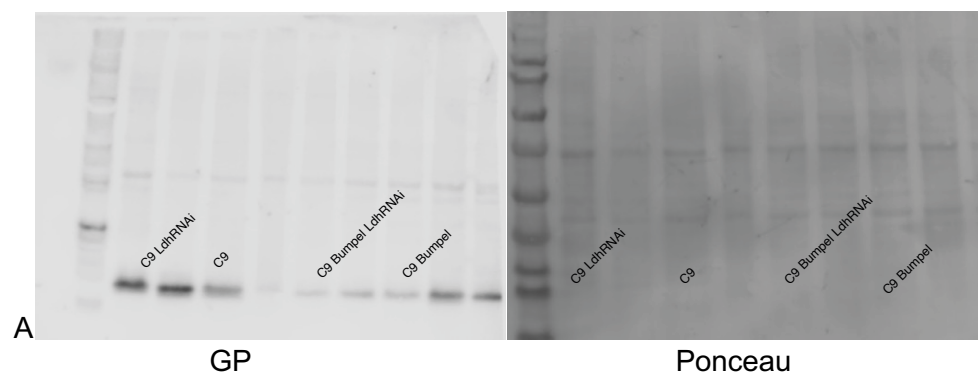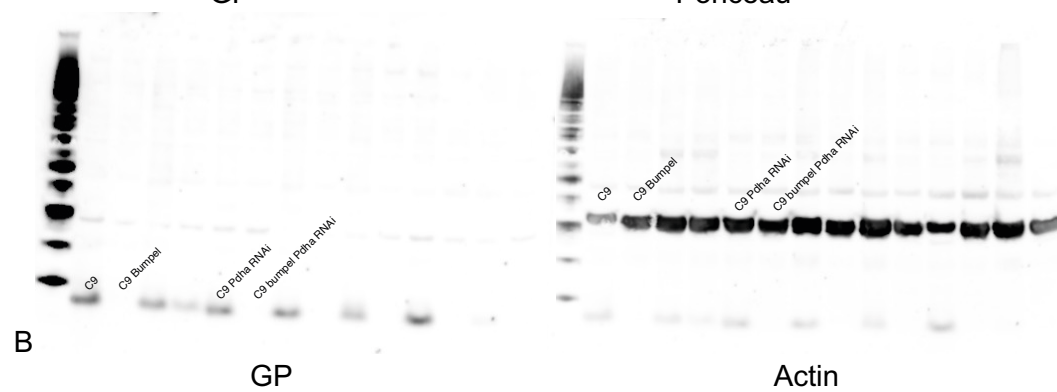

Supplement: S4 Fig — Full western blots for Fig 3D (A) and 3E (B), with samples labelled. (PDF) [file pgen.1010893.s004.pdf]

A

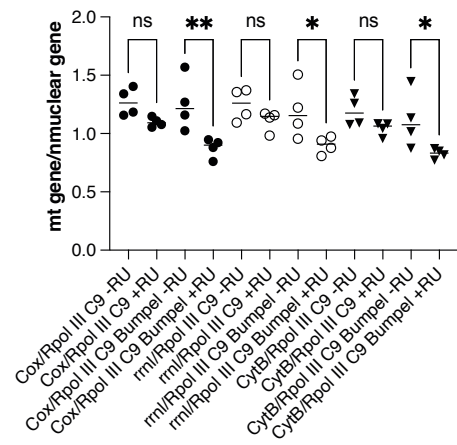

B

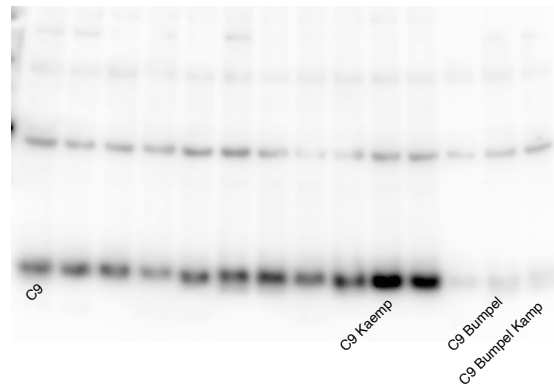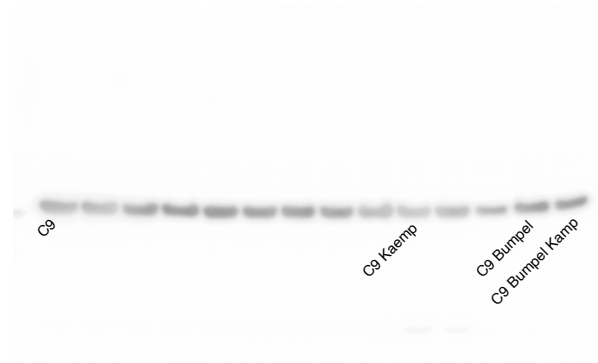

Supplement: S5 Fig — A. Mitochondrial copy-number measured by qPCR of mitochondrial DNA levels relative to nuclear DNA levels. *p<0.05, **p<0.005 by Šídák’s multiple comparisons test following one way ANOVA. +RU samples also shown in Fig 4C B. Images of the whole blots used in Fig 4F. Genotypes; UAS-36 (G4C2), elavGS, UAS-36 (G4C2), elavGS/UAS-bumpel. (PDF) [file pgen.1010893.s005.pdf]

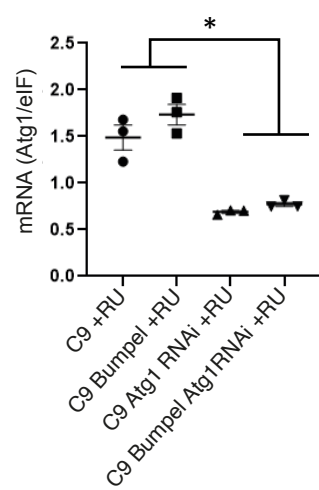

Supplement: S6 Fig — *p<0.05 by by Šídák’s multiple comparisons test following one way ANOVA. Genotypes; UAS-36 (G4C2), elavGS, UAS-36 (G4C2), elavGS/UAS-bumpel,UAS-36 (G4C2)/Atg1RNAi, elavGS, UAS-36 (G4C2)/Atg1RNAi, elavGS/UAS-bumpel. (PDF) [file pgen.1010893.s006.pdf]

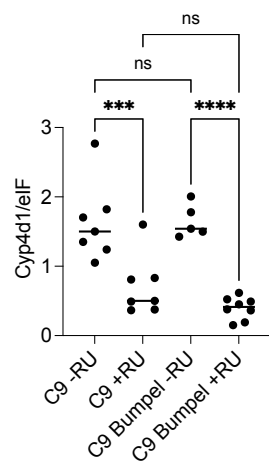

Supplement: S7 Fig — ***p = 0.001, ****p<0.0001 by by Šídák’s multiple comparisons test following one way ANOVA. Genotypes; UAS-36 (G4C2), elavGS, UAS-36 (G4C2), elavGS/UAS-bumpel. (PDF) [file pgen.1010893.s007.pdf]
